# Propolis and its constituents against cardiovascular risk factors including obesity, hypertension, atherosclerosis, diabetes, and dyslipidemia: A comprehensive review

**DOI:** 10.22038/IJBMS.2023.67793.14835

**Published:** 2023

**Authors:** Arian Khoshandam, AmirHossein Hedayatian, AmirReza Mollazadeh, Bibi Marjan Razavi, Hossein Hosseinzadeh

**Affiliations:** 1 School of Pharmacy, Mashhad University of Medical Sciences, Mashhad, Iran; 2 Targeted Drug Delivery Research Center, Pharmaceutical Technology Institute, Mashhad University of Medical Sciences, Mashhad, Iran; 3 Department of Pharmacodynamics and Toxicology, School of Pharmacy, Mashhad University of Medical Sciences, Mashhad, Iran; 4 Pharmaceutical Research Center, Pharmaceutical Technology Institute, Mashhad University of Medical Sciences, Mashhad, Iran

**Keywords:** Atherosclerosis, Cardiovascular disease Diabetes, Dyslipidemia, Hypertension, Obesity, Propolis

## Abstract

Cardiovascular diseases (CVDs) are some of the major causes of death worldwide. The modern lifestyle elevates the risk of CVDs. CVDs have several risk factors such as obesity, dyslipidemia, atherosclerosis, hypertension, and diabetes. Using herbal and natural products plays a pivotal role in the treatment of different diseases such as CVDs, diabetes, and metabolic syndrome. Propolis, a natural resinous mixture, is made by honey bees. Its main components are phenolics and terpenoid compounds such as caffeic acid phenethyl ester, chrysin, and quercetin. In this review, multiple studies regarding the pharmacological impacts of propolis and its constituents with their related mechanisms of action against mentioned CVD risk factors have been discussed in detail. Here, we used electronic databases or search engines such as Scopus, Web of Science, Pubmed, and Google Scholar without time limitations. The primary components of propolis are phenolics and terpenoid compounds such as caffeic acid phenethyl ester, chrysin and quercetin. Propolis and its constituents have been found to exhibit anti-obesity, anti-hypertension, anti-dyslipidemic, anti-atherosclerosis, and anti-diabetic effects. The vast majority of studies discussed in this review demonstrate that propolis and its constituents could have therapeutic effects against mentioned CVD risk factors via several mechanisms such as antioxidant, anti-inflammatory, reducing adipogenesis, HMG-CoA reductase inhibitory effect, inhibition of the ACE, increasing insulin secretion, NO level, etc.

## Introduction

One of the major causes of death worldwide is cardiovascular disease. Various risk factors for cardiovascular diseases (CVDs) have been established until now, such as smoking, physical inactivity, and poor diet (1).

Common manifestations of CVDs such as ischemic or transient stroke, venous thromboembolism, myocardial infarction (MI), and peripheral artery disease (PAD) are caused by thrombotic complications. Atherosclerosis, which is promoted by inflammatory and thrombotic mediators is a chronic cause of atherothrombotic events (2).

CVDs are the principal cause of morbidity and death in type 2 diabetes (T2D). The mortality rate of CVDs in T2D in women is higher than in men when compared with non-diabetic patients with CVDs. Therefore, the prevention or control of diabetes is crucial for the prevention of CVDs, especially in women (3).

CVDs are some of the most important adverse effects of obesity. It is considered a pivotal risk factor for coronary heart disease (CHD), hypertension (HTN), and heart failure (HF). In recent decades obesity became a major problem and it is predicted that failing to control the obesity epidemic could lead to a reversal of the steady enhancement in expectancy of life (4).

Many studies have revealed that hypertension is an important risk factor for CVDs. Reducing blood pressure not only prevents CVDs but also helps to manage other diseases such as brain-cardiorenal cross-talk it also decreases the risk of noncardiovascular diseases such as cancer, oral disorders, and dementia (5). One of the principal risk factors of CVDs is dyslipidemia. Studies show that dyslipidemia increases the risk of atherosclerosis. MI and stroke are associated with low high-density lipoprotein (HDL) and high-low-density lipoprotein (LDL) levels (6).

Some plants and their active constituents that could be beneficial in mentioned cardiovascular risk factors including obesity, hypertension, atherosclerosis, dyslipidemia, and diabetes include* Nigella sativa* (black seed) and its active constituent thymoquinone (7), *Persea americana* (avocado) (8), *Vitis vinifera* (grape)(9), *Rosmarinus officinalis* (rosemary) (10), *Cinnamomum verum* (cinnamon)(11), *Allium sativum* (garlic)(12), *Crocus sativus* (saffron )(13), *Berberis vulgaris* (barberry)(14), *Silybum marianum* (milk thistle)(15), *Capsicum annum* (16), *Garcinia mangostana* (17), and *rutin *(18).

Bees produce several well-known products, such as royal jelly, bee pollen, honey, bee venom, and propolis. Many of these substances have been used as food and traditional medicines (19, 20). The most famous of these compounds is honey, which is obtained from flowers’ nectar (20). Honey is used in the treatment of microbial infections (21) and stimulation of the immune system (21, 22); it also has antibacterial, anti-inflammatory, anti-diabetic, and anti-cancer impacts (22). Another compound produced by young worker bees is royal jelly which is used in the feeding of larvae and adult queens (21). It consists of water, carbohydrates, proteins, lipids, vitamins, and minerals (21, 23, 24). Royal jelly has many properties such as antimicrobial activity (21, 23), anti-cancer (24), anti-oxidant (24), anti-hypercholesterolemic, and anti-inflammatory effects (24). It has effectiveness in improving the damage of chemotherapy (23).

The word propolis means guard of the beehive (beehive as a city) (25). Hence, propolis is considered a sealant for bees (26). Propolis is a natural resinous mixture made by honey bees and it is the final product of three different sources:1-plant resins collected by bees 2 - substances secreted by bee metabolism (wax) 3-other substances (27). The chemical mixture of propolis is determined by the composition of the sources which depends on local flora (27, 28), seasons, and hives (28).

**Figure 1:**
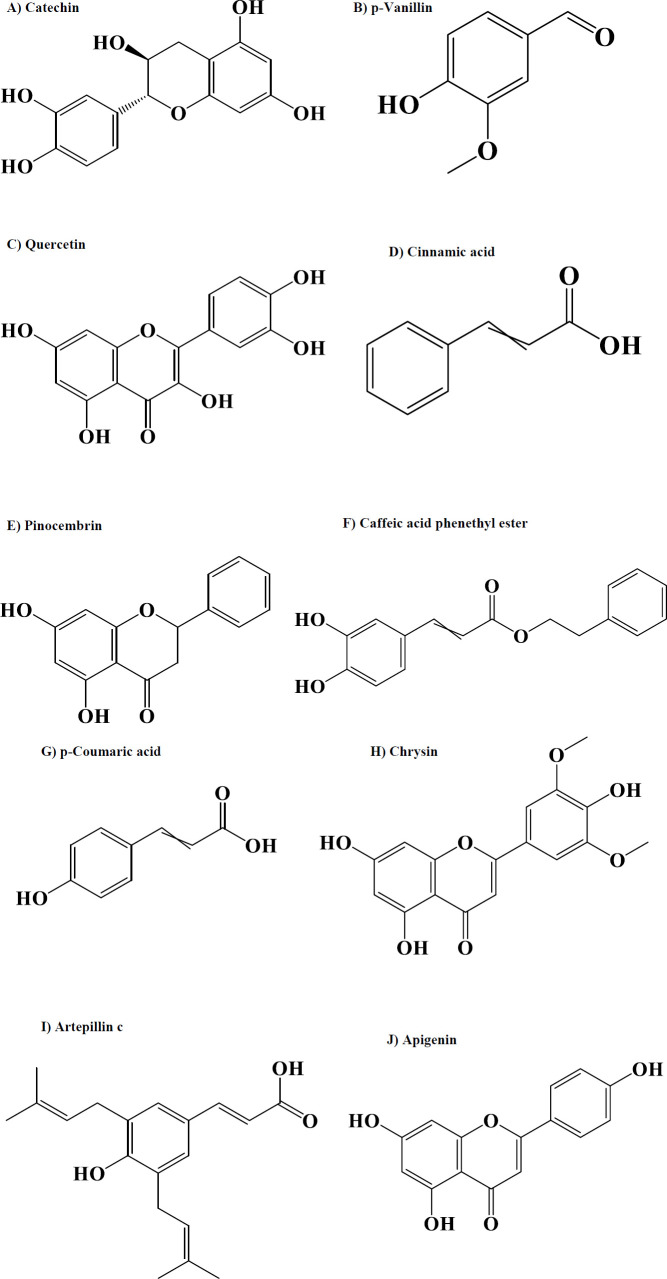
Structures of bioactive compounds from propolis

The components in propolis include phenolic acids, esters, flavonoids, diterpenes, sesquiterpenes, aromatic aldehydes, lignans, fatty acids, amino acids, alcohols, minerals, and vitamins (28). Its main components are phenolics and terpenoid compounds such as caffeic acid phenethyl ester, chrysin, and quercetin (29) (Figure 1).

From a physicochemical view, propolis is a lipophilic material (26). Propolis is hard and fragile when it is cold (25, 26) and when it is warm, it is soft, sticky, and flexible (25, 26). It has a variable color that is associated with the plant source, age, and geographical climate varying from cream, green, or red to brown (25, 26). Bees use propolis to seal gaps, coat areas in the beehive, keep humidity and temperature stable in the hive, and protect them from microorganism invaders. Moreover, they use propolis as a balsam substance to mummify irremovable invaders. Therefore, propolis is an anti-microbial agent that may invade the beehive. Propolis has been used in traditional medicine due to its anti-microbial, anti-inflammatory, anti-oxidant, immunomodulatory, anti-mutagenic, and carcinostatic properties (30). The first people who used propolis are ancient Greek, Romans, and Egyptians (25). They used propolis as a wound healer and disinfectant substance (25). Others, such as the Chinese, Indians, and Arabs used propolis to cure pathologic conditions such as skin lesions (25). It has been used in dental hygiene products such as toothpastes and cosmetics such as lotions, and also it is considered a food preservative (26).

More than 300 compounds are known in different species of propolis (31). It is pivotal to consider that propolis’ contents such as volatile compounds, which play important roles in the organoleptic effects of propolis, vary depending on geographic origin, vegetation and respective resins, climatic conditions, and the pollen types of each region (31). Propolis from Brazil, China, and Germany has the same scavenging activity against 1,1-diphenyl-2-picrylhydrazyl (DPPH) free radicals whereas Peruvian propolis has weaker scavenging activity. Propolis from Okinawa has anti-oxidants not seen in propolis from other countries such as Uruguay, Argentina, Ukraine, Bulgaria, Austria, Hungary, Brazil, China, Chile, South Africa, New Zealand, USA, Thailand, or Uzbekistan (32). While propolis is effective in CVDs, it is a mixture of natural substances such as phenolic acids, esters, flavonoids, diterpenes, sesquiterpenes, aromatic aldehydes, lignans, fatty acids, amino acids, alcohols, minerals, and vitamins (28). Therefore, it is important to do further study to determine the main mechanisms correlated to the therapeutic activities of propolis and the effective dosage with promising effects (33). Moreover, it is important to take some precautionary measures into account in case of an allergic reaction (33).

**Figure 2.**
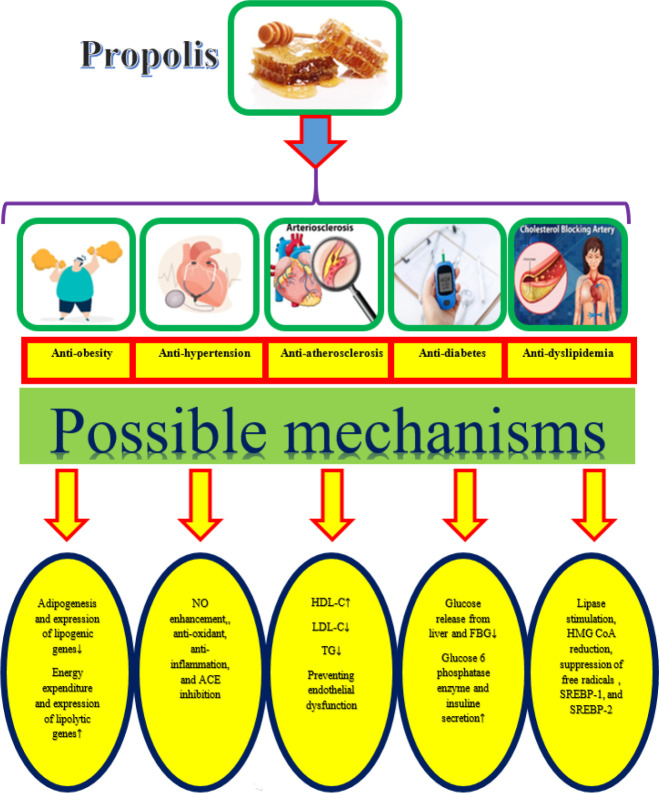
Beneficial effects of propolis and its constituents on different risk factors of cardiovascular diseases

NO: nitric oxide; ACE: angiotensin-converting enzyme; FBG: fasting blood glucose; HDL-C: high-density lipoprotein-cholesterol; LDL: low-density lipoprotein; TG: triglyceride; HMG-CoA: 3-hydroxy-3-methyl-glutaryl-CoA; SREBP-1: sterol regulatory element-binding protein 1; SREBP-2: sterol regulatory element-binding protein 2

Propolis and its constituents are effective in the treatment and prevention of cardiovascular risk factors including obesity (34), hypertension (35), atherosclerosis (34), dyslipidemia (36), and diabetes (37) (Figure 2).

In this review, we gathered various *in vivo*,* in vitro*, and human studies on the effects of propolis and its active constituents on different cardiovascular risk factors, including hypertension, obesity, dyslipidemia, atherosclerosis, and diabetes.

***Search method***

In this review, we used the electronic databases and search engines including Scopus, Web of Science, Pubmed, and Google Scholar without time constraints. The search keywords contained “Propolis,” “Blood pressure,” “Hypertension,” “Hypotensive,” “Anti-hypertensive,” “Hypertensive,” “Diuretic,” “Diabetes,” “Hyperglycemia,” “Insulin,” “Hypoglycemic,” “Blood glucose,” “Dyslipidemia,” “Hyperlipidemia,” “High cholesterol,” “Hypercholesterolemia,” “Atherogenic,” “Atherosclerosis,” “Obesity,” “Anti-obesity,” “Bodyweight,” “Food intake,” and “Feed intake”.

***Effects on obesity***

Obesity, an important problem for human health globally, is correlated with cardiovascular risk factors, such as dyslipidemia, intolerance of glucose, and hypertension (34). Obesity is a risk factor for CVDs (38). There are different *in vivo *and* in vitro* studies about the effects of propolis and its constituents on obesity that are mentioned in this review.

***In vivo studies***

Mice were given a high-fat diet (HFD), and HFD plus ethanolic extract of propolis(EEP)(50 mg/kg for 30 days). HFD plus EEP group of mice showed lower of body weight gain compared with the other groups (HFD and control). The possible mechanisms of EEP to decrease weight and weight gain are suppressing adipogenesis and elevating the expenditure of energy (34).

In another experiment, the mice were divided into three groups (control, high-fat (HF)-diet-fed, and HF-diet-fed plus poplar propolis ethanolic extract (PPEE)). The results showed that PPEE (20 mg per mouse per day for 12 weeks) significantly diminished the increase in body weight caused by HF diet. The intake of energy was greater in the HFD-fed group in comparison with the control group, but there was no remarkable difference in the PPEE-supplemented group in comparison with the HF-diet-fed group. The suggested mechanism was decreasing relative and absolute fat mass in inguinal fat pads, epididymal, and retroperitoneal in PPEE-fed mice, in comparison with the HFD-fed mice. There was no remarkable difference in adiposity index between PPEE-fed mice and control-fed mice (39).

According to another study, four-week-old Wistar rats were fed an HFD or propolis (0.05% and 0.5%) diet for 8 weeks. The weight of white fat tissue of the rats that had propolis in their diet was lower than the HFD group. Peroxisome proliferator-activated receptor gamma (PPARγ) has a pivotal role in the fat tissue induced by an HFD. The reason for decreasing the accumulation of fat could be the reduction of the level of PPARγ protein by propolis (40).

Koya-Miyata *et al*., evaluated the effects of propolis on obesity. Mice were fed a high-fat diet and given propolis at 5 mg/kg or 50 mg/kg twice daily for 10 days. Administration of propolis to the mice caused reduction of weight of visceral adipose and body weight gain. The proposed mechanisms were the reduction of sterol regulatory element-binding protein 1 (SREBP-1), sterol regulatory element-binding protein 2 (SREBP-2) mRNA levels, and gene expression of fatty acid synthase (FAS) and acetylacetonate anion (ACAC) activated by SREBP-1. Therefore the possible mechanism could be the reduction of fatty acid synthesis receptors (41).

The effects of 2.3 g/kg Brazilian green propolis supercritical extract (GPSE) in the female C57BL/6NRj wild-type mice on body weight and food intake have been studied for ten weeks. GPSE-CD did not affect the body weight and food consumption of the mice (42).

In a study, the anti-obesity effect of CAPE which is a major part of poplar-type propolis was shown. Mice were fed an HFD supplemented with CAPE (0.02, 0.1, or 0.5%) for 5 weeks. HFD-fed mice demonstrated a higher level of adiposity. Supplementation of CAPE decreased the level of adiposity in mice. CAPE reduced mRNA expression of lipogenic and adipogenic genes such as C/EBPα, PPARγ, and FAS, and elevated mRNA expression of thermogenic and lipolytic genes such as adipose triacylglycerol lipase (ATGL), peroxisome proliferator-activated receptor-gamma coactivator 1α (PGC-1α), and long-chain acyl CoA synthetase (ACSL). These results suggest that CAPE decreases body weight gain caused by HFD and adipogenesis and increases energy expenditure (43).

Nishikawa *et al*., evaluated the anti-obesity effects of Artepillin C (ArtC) (Figure 1), a typical component of Brazilian green propolis in C57BL/6J mice (doses: 5 mg/kg and 10 mg/kg for 28 days). Brown-like adipocytes induce thermogenesis associated with UCP 1 and with this mechanism can reduce body fat accumulation. ArtC induces brown-like adipocytes. Thus, propolis could be beneficial in preventing and treating obesity (44).

In another study, the anti-obesity effect of chrysin, a major component in poplar-type propolis, in six groups of rats including control, HFD control, orlistat, and chrysin (25, 50, and 100 mg/kg bodyweight for 16 weeks) has been evaluated. Chrysin demonstrated an inhibitory effect on pancreatic lipase (PL). Chrysin could also significantly reduce acute feed consumption in rats. Chrysin significantly reduces the body mass index (BMI), bodyweight, abdominal circumference/thoracic circumference (AC/TC) ratio, and adiposity index. Therefore, chrysin has anti-obesity effects (45).

According to another experiment, the effects of propolis on body weight gain have been evaluated. Western diet caused obesity in mice. Propolis was administered to C57BL/6N mice for 8 weeks. The propolis diet group had less body weight gain compared with the western plus propolis group. The suggested mechanisms were down-regulation of the lipid metabolism genes and inhibiting the formation of adipose tissue by suppressing the extracellular-signal-regulated kinase (ERK) activity (46).

***In vitro studies***

Orlistat is a famous medication that leads to weight decrease in obese people. In an experiment, the anti-obesity and inhibitory effects of Australian propolis compounds (83 μM) versus orlistat on lipase have been investigated. Orlistat and Australian propolis compounds showed anti-obesity properties but orlistat demonstrated 6-10 times more lipase inhibitory effects compared with Australian propolis compounds (47). 

The anti-obesity effects of the ethanolic extracts of red propolis (EERP) at the concentrations of 0-30 μg/ml in an experiment performed on 3T3-L1 cells for ten days have been shown. This study showed that EERP significantly enhances 3T3-L1 adipocyte differentiation which is followed by a high induction of genes that are necessary for the differentiation of adipocytes such as adiponectin. The EERP impacts on adipogenesis of 3T3-L1 cells were demonstrated in part through PPARγ activation. EERP reversed the inhibitory effects of tumor necrosis factor-α (TNF-α) on adiponectin expression and adipocyte differentiation. Thus, EERP could be an effective diet supplement for the treatment and prevention of obesity and obesity-related disorders such as MetS and CVDs (48).

In another study, the effects of CAPE in the stem cells-derived adipocytes from a healthy patient (male, 23 years old, 98 kg body weight) on adipogenesis were shown. After 14 days, lipid droplets were reduced following CAPE (10 μM) treatment. This result was verified by a significant decrease in reactive oxygen species (ROS) formation caused by CAPE. Moreover, the expression of the anti-oxidant enzyme HO-1 following CAPE treatment was significantly enhanced after 14 days. Thus, CAPE reduces lipogenesis and could be effective in preventing and treating obesity (49).

Nishikawa *et al*., investigated the anti-obesity effects of ArtC in C3H10T1/2 cells. ArtC induces brown-like adipocytes due to activation of PPARγ (44).

According to another experiment, the effects of chrysin (25, 50, and 100 mg/kg bodyweight for 16 weeks) on adipocytes have been investigated. Chrysin reduced the adipocyte size in comparison with the HFD control rats (45).

***Human studies***

In an experiment on 34 healthy volunteers, oral administration of bee propolis at a dose of 1000 mg daily for 60 days, caused remarkable body weight gain. The appetite of these volunteers was enhanced after propolis administration and this could be a possible mechanism for its effect on body weight. Another suggested mechanism could be the elevated level of hepatic glycolysis and uptake of glucose by tissues through insulin-sensitive glucose transporter enhancement after propolis administration (50).

According to the studies, propolis has anti-obesity effects and prevents weight gain in obese mice by different mechanisms such as inhibition of adipogenesis, increased energy expenditure, reduction of absolute and relative fat mass, reduction of the level of PPARγ, suppressing the ERK activity, increasing the expression of thermogenic and lipolytic genes, etc. Interestingly, based on some discussed papers in this review, propolis could prevent weight loss through possible mechanisms such as appetite and hepatic glycolysis enhancement (Table 1).

***Effects on hypertension***

Hypertension has harmful effects on various organs such as the brain, kidneys, heart, and vascular systems. It is one of the major risk factors in the pathogenesis of CVDs and increases mortality and morbidity (51). Propolis and its constituents are effective in treating and preventing hypertension. There are different *in vivo*,* in vitro*, and human studies about the effects of propolis and its constituents on hypertension that are mentioned in this review.

***In vivo studies***

In a study, the anti-hypertensive effects of propolis (200 mg/kg/day for 28 days) and CAPE on hypertensive Spraque-Dawley rats have been studied. The rats treated with CAPE and propolis had lower blood pressure than the hypertensive group. The tyrosine hydroxylase (TH) and catecholamine levels increased in hypertensive rats. The suggested mechanism was decreased level of TH and inhibited biosynthesis of catecholamine by CAPE and propolis (52).

In another experiment, the effects of flavonoids from Brazilian green propolis (10 mg/kg, orally for 28 days) such as dihydrokaempferide, isosakuranetin, kaempferide, and betuletol in spontaneously hypertensive rats (SHR) have been studied. Dihydrokaempferide, isosakuranetin, and betuletol decreased systolic blood pressure in SHR but the effect of kaempferide was not significant. The anti-hypertensive effect of these flavonoids may be due to vasodilatory effects (53).

Salmas *et al*., evaluated the effects of propolis in Nωnitrolarginine methyl ester (L- NAME) induced hypertensive male Wistar rats. Propolis (200 mg/kg) was administered during the last 5 days of the 15 day experiment. The propolis-treated hypertensive rats showed lower hypertension compared with the L-NAME-induced hypertensive group by enhancing the level of nitric oxide (NO) (54). Also, the effects of CAPE (50 μM/kg/day) and propolis (200 mg/kg/day by gavage) in L-NAME-induced hypertensive male, Sprague Dawley rats have been studied. The agents were given on the last 14 of 28 days. Propolis and CAPE showed anti-hypertensive effects by decreasing the level of asymmetric dimethylarginine (ADMA) and nuclear factor-kappa b (NF‐κB). So, propolis and CAPE increased the level of NO (55).

In another experiment, the effects of chrysin (25 mg/kg for 4 weeks) in L-NAME-induced hypertensive male albino rats have been studied. Administration of chrysin significantly reduced the thiobarbituric acid reactive substances (TBARS) levels, conjugated dienes, and lipid hydroperoxides, and markedly enhanced the catalase (CAT) activity, superoxide dismutase, glutathione peroxidase, decreased glutathione, vitamin E, and vitamin C in the circulation and tissues in comparison with the L-NAME induced hypertensive group. Inhibiting superoxide anions by chrysin is related to its anti-oxidant effect and leads to vessel wall relaxation and controls blood pressure (56).

Anti-hypertensive effects of propolis in L-NAME-induced hypertensive male Wistar rats have been shown in another study. Propolis (200 mg/kg) was given by gavage for the last 5 of 15 days. Treated rats with propolis showed lower malondialdehyde (MDA), CAT activity, and ROS levels compared with the control group. The anti-hypertensive mechanism may be due to anti-oxidant effects because of the presence of many phenolic compounds and amino acids with anti-oxidant properties. Moreover, rats that had propolis in their diet, demonstrated a greater level of NO in comparison with the L-NAME-induced hypertensive rats (57).

According to another experiment, the anti-hypertensive properties of Chinese water-soluble propolis (WSP) in the high-salt-fed induced hypertensive rats at 100 mg/kg for the last 4 of 14 weeks have been studied. WSP enhanced the level of endothelial nitric oxide synthase (eNOS) and NO. Thus, it showed the arterial relaxation effect. Furthermore, WSP decreased the level of pro-inflammatory cytokines such as IL-6 and TNF-α. Moreover, it decreased the levels of Nox2, Nox4, and ROS in high salt-induced hypertensive rats (35).

The effects of propolis (200 mg/kg/days, gavage for 28 days) in L-NAME-induced hypertensive rats in another study have been demonstrated. The MDA level, NF-κB, and total oxidant status were lower in the L-NAME plus propolis group in comparison with the L-NAME group. But the level of CAT, paraoxonases (PON1), total anti-oxidant status, and NO level have increased in the L-NAME plus propolis group in comparison with the L-NAME group. So the antihypertensive effects of propolis were attributed to its anti-oxidant, anti-inflammatory, and vasodilatory effects (58).

The preventive effects of propolis (200 mg/kg for 4 weeks) in high-salt diet-induced hypertensive rats were evaluated. Propolis diminished both systolic and diastolic blood pressures. The anti-hypertensive mechanism of propolis may be due to the metabolism of the lipids. The low-density lipoprotein (LDL) level was lower in groups that received propolis from Riau Archipelago and propolis from Lampung. LDL level is correlated with hypertension due to its association with leptin resistance. The HDL level was higher in the groups that received propolis from Riau Archipelago and South Sulawesi compared with the high-NaCl-only group. Low HDL levels may correlate with hypertension due to its association with reducing kidney function. Another possible suggested mechanism was the diuretic effect of propolis from Riau Archipelago and Lampung by vasodilation and removing any extra fluid. This study demonstrated that propolis has anti-hypertensive effects (59).

***Human studies***

In a randomized controlled trial study, the anti-hypertensive effects of propolis have been shown. Propolis (3%) was administered to a human population (n=32) in Talca for 90 days. Propolis notably decreased both systolic and diastolic blood pressures. Phenolic and flavonoid compounds, such as cinnamic acid, quercetin, p-vanillin, p-coumaric acid, and apigenin, are all present in Chilean propolis neutralized oxidative species. CAPE activated the transcription factor Nrf2 which is involved in the production of anti-oxidant mediators. Thus, the anti-hypertensive effect of propolis may be due to the anti-oxidant and anti-inflammatory effects (60).

Thus, according to the studies, propolis and its constituents are effective in reducing hypertension through mechanisms such as increasing the level of NO, vasodilation, ACE inhibitory effects, decreasing the level of oxidative stress, asymmetric ADMA, etc. (Table 2). 

***Effects on atherosclerosis***

Atherosclerosis develops in medium and large-sized arteries and is considered a chronic inflammatory disease (61). It is the principal cause of coronary artery disease and stroke (62) with a high mortality rate in most populations (63). Propolis and its constituents are effective in curing or reducing the pathology of atherosclerosis. There are different *in vivo *and* in vitro* studies about the effects of propolis and its constituents on atherosclerosis that are mentioned in this review.

***In vivo studies***

The ethanolic extracts of propolis (EEP) (50 mg/kg for 30 days) elevated the level of HDL-C in comparison with the male mice fed only with HFD and had smaller LDL-C and triglycerides levels. EEP also elevated plasma HDL-C levels in normolipidemic mice. High HDL neutralizes the atherogenic effect of ox-LDL in the artery wall (34). A cholesterol-rich diet increases oxidized LDL (Ox-LDL) which appears to contribute to atherosclerosis pathobiology (64). The ethanolic extract of propolis (50 mg/kg for 30 days) exhibits an anti-atherosclerosis effect in mice treated with HDF via reducing the oxidation of LDL due to the activation of the transcription factor NrF2 and improvement of the anti-oxidant enzymes including phase II detoxification enzymes, heme oxygenase-1, and enzymes associated with the metabolism of GSH. The other mechanism is the elimination of oxidant species which prevents the activation of the NF-κB signaling pathways (34). EPP (160 mg/kg/day for 14 weeks) remarkably diminished the level of total cholesterol (TC), triglyceride (TG), and non-HDL-C in ApoE−/− mice fed HFD. It also reduces the level of IL-6 and IL-17, which may be one of the mechanisms that prevent the progression of atherosclerosis in its early stage. IL-6 which is secreted from mononuclear macrophages has moderate inflammatory activity and is highly related to atherosclerosis and its development (65). The imbalance of endothelin (ET) and NO is one of the causes of endothelial dysfunction. ET-1 is a dominant molecular form of endothelium effective in atherosclerosis development. NO might prevent all of the main processes in the formation of early pathogenesis. Inducible NOS (iNOS) found in the early stages of atherosclerosis leads to nitration of NO and induction of atherosclerosis (65). EPP (160 mg/kg/day for 14 weeks) decreased the serum level of the ET while increasing the level of NO and inhibited iNOS not significantly in ApoE−/− mice fed an HFD. This observation shows the potential of propolis in reversing the imbalance between NO and ET (65). In unstable coronary plaques, there is an increase in neovascularization which is probably induced by vascular endothelial growth factor-A(VEGF-A). EEP (160 mg/kg/day for 14 weeks) markedly diminished the levels of serum VEGF in ApoE−/− mice fed an HFD (65).

In another study, the effect of EEP (150 mg/kg for 4 weeks) in mice injected with H-cholesterol-labeled and ox-LDL-loaded Raw264.7 cells has been evaluated. It has been found that EPP improves reverse cholesterol transport (RCT) from intraperitoneal macrophages to feces. Moreover, EPP stimulates the expression of ATP-binding cassette transporter A1 (ABCA1) and ATP-binding cassette transporter G1 (ABCG1) in Raw264.7 cells exposed to EEP (0, 0.2, 1.0,5.0 μg/ml/ for 48 hr). Both of these transporters are involved in cholesterol efflux from peripheral tissues to plasma. It has been suggested that an enhancement in expression of ABCA1 and ABCG1 and an increase in HDL-C level are the mechanisms by which EEP enhances reverse cholesterol transport (62).

In a study conducted on the evaluation of the effect of polyphenols of Brazilian green and red propolis and Chilean brown propolis (250 mg/kg per day for 4 weeks) on initial and advanced atherosclerosis lesions in LDLr(-/-) knockout mice, researchers found that all of the three types of propolis reduced the atherosclerotic lesion area in initial atherosclerotic lesions (IAL) group by down-regulating the excretion of CD36, a scavenger receptor contributor to the formation of foam cells, and by reducing total cholesterol and elevating the HDL-C concentration which may be related to the up-regulation of ABCA1 and CD36 (63). In the development of atherosclerotic lesions, monocyte-derived macrophages filled with atherogenic lipoproteins penetrate the subendothelial layer of the intima, which results in the build-up of lipid-laden macrophages (foam cells) (61). In the advanced atherosclerotic lesions group, only red propolis reduced the development of atherosclerosis. All three types of propolis modulate the expression of proinflammatory cytokines in both groups and all three types increase the up-regulation of HO-1 expression, which has a pivotal role in inflammatory repair. However, down-regulation of TGF-β and vascular cell adhesion molecule (VCAM) was only observed in the IAL group (63).

In another study conducted on LDLr(-/-) knockout mice, Chilean propolis (250 mg/kg) regulated the expression of Hif-1 and VEGF-A, two main elements in neovascularization that affect advanced atherosclerotic plaques. In advanced atherosclerotic lesions, inadequate oxygen diffusion from the lumen causes hypoxia because of the establishment of a necrotic core and the subsequent increase in the diameter of the arterial wall hypoxia increases the expression of genes containing Hif-responsive elements such as VEGFA by increasing the stabilization of Hif1α and Hif2α and its translocation to the nucleus (66).

***In vitro studies***

In a study, the effect of propolis on human umbilical cord endothelial cells (HUVECs) exposed to ox-LDL (45 μg/ml) has been evaluated. It has been found that propolis (12.5 mg/ml) reduces the level of ox-LDL and subsequently decreases the level of ROS which results in the prevention of NF-κB activation (67).

Ox-LDL is a strong ROS inducer. An elevation in the production of ROS leads to oxidative stress and this can lead to endothelial dysfunction and induce the progression of several diseases such as atherosclerosis by initiating downstream signal molecules such as NF-κB (67).

Macrophages have a principal role in starting the progression of atherosclerotic lesions by the uptake of ox-LDL. Therefore, macrophages could be one of the effective targets for atherosclerosis treatment (61). In one study, the effect of propolis (20 μg/ml) on macrophages treated with ox-LDL has been evaluated. Propolis prevented the formation of foam cells by suppressing the cellular uptake of ox-LDL. It also reduced the formation of cholesterol ester (CE). The increased CE in macrophages enriched with ox-LDL is likely through the process of intracellular lipid metabolism which is affected by TNF-α and IL-1β. Propolis inhibits mitogen-activated protein kinase (MAPK) phosphorylation which ultimately inhibits the secretion of proinflammatory cytokines and allows intracellular lipid catabolism to be done normally (61).

Taken together, propolis and its constituents have anti-atherosclerosis effects through the mechanisms such as anti-oxidant, reversing cholesterol transfer, changes in lipid profile, anti-inflammatory, reversing the imbalance between NO and ET, reducing neovascularization, reducing endothelial dysfunction, etc. (Table 3).

***Effects on lipid profile***

Dyslipidemia is defined as a low level of HDL and a high level of LDL, TG, and TC (68). Nowadays, new synthetic drugs have been approved for lowering blood lipids such as colesevelam, bile acid sequestrants, and statins (69). However, severe side effects of these drugs have been reported, including rhabdomyolysis and polyneuropathy (69). It is important to find a substance to reduce blood lipids from natural resources and plants (69), such as *Crataegus pinnatifida *(Chinese hawthorn)(70)*,* *Persea Americana *(avocado)(8), and* Silybum marianum *(milk thistle)(15). There are different *in vivo*,* in vitro*, and human studies about the effects of propolis and its constituents on lipid profiles that are mentioned in this review.

***In vivo studies***

In a study, the hypocholesterolemic and hypolipidemic effects of propolis in rats treated with high cholesterol diets (HCD) have been examined. Animals were assigned into four groups. The first group was fed the basal diet, the second group was fed the basal+cholesterol diet (1%). Groups 3 and 4 were treated with propolis powder 1 and 2% mixed with high cholesterol diet (1%) for 2 weeks. Findings showed that the supplementation by propolis in HCD decreased serum total cholesterol and triglycerides without any effect on HDL-C, LDL-C, and VLDL-Cc concentrations. The lack of dramatic change in the HDL-C level may be related to the fact that propolis might not interact with HDL-C synthesis. It may happen by the inhibition of 3-hydroxy-3-methyl-glutaryl-CoA (HMG-CoA) reductase, which mediates the first step in cholesterol biosynthesis (71)whereas rats of group 2 were fed basal diet mixed with cholesterol (1%.

Four-week-old Wistar rats were divided into three groups: Control group (fed an HFD), Low group (fed an HFD+0.5% (w/w) propolis), and the high group (fed an HFD+0.05% (w/w) propolis). In the liver and plasma, the levels of cholesterol and TG of the high group had a dramatic reduction in comparison with the HFD group. Administration of propolis enhanced PPARα protein and decreased SREBP-1 protein in the liver. Therefore, changes in these proteins may reduce the level of TG in the liver and plasma. Also, the high group had a markedly lower liver HMG-CoA reductase enzyme than the HFD group. The administration of propolis may ameliorate dyslipidemia and accumulation of body fat by changing the expression of proteins involved in lipid metabolism and adipose depot (40).

In another study, the protective role of Turkish *Castanea sativa *propolis against alcohol-induced oxidative stress was evaluated. Male rats were given ethanolic propolis extract (200 mg/kg/day). LDL levels were raised and HDL levels reduced in the alcohol group, while LDL levels diminished in the propolis plus alcohol group. Cholesterol and TG levels also diminished in the alcoholic plus propolis group. This study showed that Turkish *C. sativa *propolis has an anti-oxidant effect (72).

In another study, the synergistic effect of propolis and several cholesterol-lowering substances has been investigated. Rats were divided into five groups: 1% HCD, 1% HCD+Simvastatin (SVS) (10 mg/kg), 1% HCD+mixtures of selected functional foods (MSFF) (50 mg/kg), 1% HCD+MSFF (100 mg/kg), and 1% HCD+MSFF (200 mg/kg). MSFF was composed of propolis, bee pollen, *Ginkgo biloba*, nattokinase (fermented soybean), oat fiber, garlic, and red yeast rice extract. Results demonstrated 1% HCD group had a dramatic rise in TC and LDL levels in comparison with the normal group. MSFF at different doses led to a reduction of LDL and TC levels in comparison with the 1% HCD group. There was no dramatic difference in the levels of LDL and TC in rats treated with SVS at 10 mg/kg in comparison with animals treated by MSFF. However, MSFF at different doses induced no difference in HDL and TG levels in comparison with the 1% HCD group (69).

One study investigated the cardioprotective and anti-oxidant properties of Malaysian Propolis (MP) in isoproterenol- (ISO-) induced myocardial infarction in male Wistar rats. Animals were pretreated with an ethanolic extract of MP (100 mg/kg/day) for one month. Injection of ISO (85 mg/kg in saline) subcutaneously for two consecutive days resulted in a dramatic enhancement in serum lipid profiles. A notable enhancement in levels of TC, VLDL-C, and TG and a reduction in HDL-C levels were seen in ISO-treated animals. Also, pretreatment with propolis exhibited a dramatic decrease in the levels of TG, TC, and VLDL-C while raising the HDL-C level in comparison with the ISO-treated group. MP reduced TC levels and increased HDL-C levels, which may be related to the up-regulation of ABCA 1 gene expression associated with increased HDL-C levels and restoration of lipid profiles in animals. Administration of MP alone has no remarkable impact on the lipid profile compared with the control group (36).

Another study evaluated the effects of chrysin (natural flavonoid ingredient of honey and propolis)against age-associated inflammation, hyperglycemia, and hypercholesterolemia. Chrysin (20 mg/kg) was injected intraperitoneally (IP) to male Wistar rats for one month. Increased glucose, lipid profile, and cytokines in the sera of aged rats in comparison with the young rats were shown. Serum levels of TG, TC, and LDL-C in the aged animals had a significant rise in comparison with the young ones. Also, a decrease in HDL-C levels in 20-month-old rats compared with 2-month-old ones was seen. 20-month-old rats treated with chrysin had a notable reduction in the serum level of TC and a dramatic elevation in the HDL-C level in comparison with the non-treated age-matched group (73).

In another experiment, the effects of propolis on metabolic derangement induced by rifampicin/isoniazid medications have been examined. Sixty four rats were divided into eight groups. Every eight rats were treated with the specific medications and doses on daily basis: the positive control group received propolis only, the negative control group received normal saline, and the isoniazid group and the rifampicin group received isoniazid or rifampicin or a combination of them with/without propolis. After 8 weeks of continuous drug administration, a dramatic decrease in TG and TC, and elevation of HDL was observed (74).

In another study, the improving role of CAPE, a substance extracted from propolis, on insulin resistance was examined. In this experiment, there were normal and non-insulin-dependent diabetic (NIDDM) groups. NIDDM mice were fed an HF diet and injected with a dose of streptozotocin (90 mg/kg) to induce partial insulin deficiency. Then this group was divided in to HF+CAPE 15 mg/kg, HF+CAPE 30 mg/kg+HF control. Treating mice with CAPE for five weeks ameliorated hyperlipidemia, insulin sensitivity, and PPARα levels. A notable rise in TG, TC, and LDL-C levels and a reduction in serum levels of HDL-C in the HF group were seen compared with the normal group. By administration of high-dose CAPE, these parameters were dramatically alleviated. PPARα can modulate inflammatory pathways and lipid profiles. Although PPARα does not have a direct effect on plasma glucose or insulin levels in diabetic subjects, PPARα agonist combined with an anti-diabetic agent benefited both lipid and glucose metabolism in T2D. The gene expression of TNF-α in tissues was significantly diminished in CAPE-treated mice (75).

***Human studies***

The effects of bee propolis on serum lipid profile, insulin resistance, and glycemic control indices in patients with type T2D were assessed in this study. Sixty-six patients with T2D were divided into two groups: intervention (IG) and placebo (PG). For twelve weeks, IG was treated with 300 mg of propolis pills three times a day, whereas PG received similar pills without propolis. A dramatic change in TC and LDL between the two groups was seen that revealed the protective properties of propolis against TC and LDL rise. No dramatic differences in other lipids between IG and PG were observed. Based on this study, the daily intake of 900 mg of bee propolis led to some serum lipid amelioration in patients with T2D (76).

Based on the above-mentioned studies, it has been concluded that administration of propolis and its components resulted in a remarkable decrease in TC, TG, and LDL-C levels and a rise in HDL-C levels. The proposed mechanisms are the inhibition of HMG-CoA reductase, which is a rate-limiting enzyme of cholesterol biosynthesis, change of the expression of proteins involved in lipid metabolism and adipose depot, anti-oxidant effect, up-regulation of ABCA 1 gene expression, etc. (Table 4). 

***Effects on diabetes***

Diabetes is an endocrine system disorder that has negative effects on the cardiovascular system (77, 78). Today, the rate of diabetes in the world is increasing rapidly for various reasons, such as reduced mobility and an unhealthy diet (79). According to WHO, the economic, financial, and human costs of diabetes are high (80). Although different types of anti-diabetic drugs (such as metformin) are used today to treat and reduce the symptoms of diabetes, still there are not any more effective ways to prevent the development of this disease (79). Currently, studies have been conducted to find substances of natural origin to treat or reduce the symptoms of diabetes, such as *Allium sativum *(81) and *Garcinia mangostana* (82). There are animal and human studies that show propolis can also reduce the symptoms of diabetes. 

***In vivo studies***

Malaysian propolis (300 mg/kg for four weeks) with or without metformin improves hepatic anti-oxidant status in streptozotocin-induced diabetic rats by increasing hepatic CAT, GPx, SOD, glutathione reductase (GR), and glutathione-S-transferase (GST) activities, and drastic elevation of GSH levels and total anti-oxidant capacity. It also significantly decreased the MDA level. Malaysian propolis may improve hepatic anti-oxidant status (83).

Chrysin (40 mg/kg BW for sixteen weeks) attenuated the diabetic nephropathy in HFD/STZ-induced type 2 diabetic rats via recovery of renal function, pathology, renal inflammation, and renal oxidative stress by suppressing TNF-α expression. An increase in TNF-α level leads to altered glomerular filtration rate (GFR), which promotes renal injury in diabetic kidneys. Moreover, Chrysin inhibits NF-кB, which is one of the transcription factors promoted by TNF-α. NF-кB controls several genes that have a vital role in diabetic nephropathy pathogenesis, like that encoding TGF-β, chemokine ligand2 (CCL2), and intercellular adhesion molecule (ICAM). TGF-β elevates the expression of extracellular matrix (ECM) proteins resulting in their build-up in tubular and mesangial epithelial cells in diabetic kidneys, which changes renal morphology (84).

Another study examined the beneficial role of propolis against DM-induced impaired pregnancy outcomes and placental oxidative stress with bigger impacts when combined with insulin. Forty female rats were divided into 5 groups: non-DM, DM, DM+Propolis orally; DM+Insulin SC, and DM+Combined (propolis+insulin). For 4 weeks, the rats were treated with propolis and insulin at 5.0 IU/kg/day SC and 300 mg/kg/day orally. The DM group had a markedly higher FBS level than the non-DM group. There was a dramatic improvement in FBS in animals who received propolis or insulin alone with more dramatic amelioration in animals treated with both insulin and propolis. This suggests the ability of insulin and propolis to decrease placental oxidative stress (85).

In another study, the anti-oxidant properties of EEP on kidneys in diabetic male Wistar rats were evaluated. Forty animals were assigned into five groups: control, DM, DM with propolis (100 mg/kg), DM with propolis (200 mg/kg), and DM with the vehicle. STZ (60 mg/kg) was injected IP to induce DM in animals. For six weeks diabetic groups received a vehicle or EEIP. The results demonstrated that Iranian propolis dramatically inhibits BW loss in diabetes mellitus rats. The propolis extracts markedly decreased kidney weight and serum glucose levels in diabetic animals. Desirable effects of the propolis are mediated by decreasing blood glucose levels in diabetic animals. Propolis contains anti-oxidants such as flavonoids and polyphenols and they decrease blood glucose levels (86).

Another study investigated EEMP hypoglycemic effect in diabetic female rats. In the 4-week study, animals were divided into five groups: Non-DM, DM, DM plus metformin (100 mg/kg/day), DM plus 300EEP (300 mg/kg/day), and DM+600EEP (600 mg/kg/day). STZ (60 mg/kg) was injected IP to induce DM in animals. FBG and total food intake were markedly higher whereas body weight gain was dramatically lower in the DM group in comparison with the Non-DM one. Diabetic rats treated with 600EEP had dramatically less total food intake in comparison with the DM group. Lower fasting blood glucose and higher body weight gain were observed in DM+metformin, DM+300EEP, and DM+600EEP groups in comparison with the DM group. EEP decreased total food intake and FBG level and raised BW gain in STZ-induced diabetic female rats. The protective effect of the propolis bioactive components on pancreatic beta cells may reduce the blood glucose level (87).

In another study, the effect of Moroccan propolis on blood glucose was examined in diabetic rats. Diabetes was induced by STZ. For fifteen days, the rats received glibenclamide, distilled water, or propolis extract (50 or 100 mg/kg) daily. Blood glucose was assessed on day 15 after the beginning of the treatment. A dramatic decrease in blood glucose after a single administration of propolis or glibenclamide and at day 15 after daily administration was seen in diabetic animals. Both interventions resulted in a notable reduction of lactic acid dehydrogenase, a rise in body weight, and an improvement of dyslipidemia and abnormal kidney and liver function caused by diabetes. The effect of propolis was dose-dependent and it was more effective than glibenclamide in a high dose. Propolis has strong antihyperlipidemic, antihyperglycemic, and hepato-renal therapeutic roles in diabetes. It has potent anti-oxidant activity. The hypoglycemic effect of propolis might be through the high amount of flavonoids and phenols content and suppression of α-amylase and α-glucosidase activities that delay glucose absorption by the intestine (37).

***Human studies***

A study evaluated the effects of Chinese propolis (CP) on inflammatory cytokines, anti-oxidant function, and glucose metabolism in patients with T2D. According to fasting serum glucose levels at the baseline, 61 T2D patients were assigned to a control group and a Chinese propolis group (900 mg/day). At the end of this 18-week study, serum glucose, glycosylated hemoglobin, insulin, aldose reductase, or adiponectin of these groups had no notable difference. However, a marked increase in serum GSH and a dramatic reduction in serum lactate dehydrogenase activity were observed in the Chinese propolis group. In conclusion, CP can enhance anti-oxidant activity in T2D patients, partially by raising serum anti-oxidant parameters (88).

In another study done on diabetes patients (30-55 years of age), the total anti-oxidant capacity, SOD, and GPx were elevated remarkably after supplementation with propolis (1500 mg/d for 8 weeks) which results in insulin resistance reduction (89).

Another study evaluated the effect of Iranian propolis extract on glucose metabolism, insulin resistance, renal and liver function, and inflammatory biomarkers in patients with T2D. In this 90-day study, patients with T2D were divided into a placebo group (n=44) and an Iranian propolis group (1000 mg/day) (n=50). A dramatic reduction was seen in parameters such as the serum levels of insulin, glycosylated hemoglobin (HbA1c), 2-hour postprandial (2hpp), homeostasis model assessment-insulin resistance (HOMA-IR), homeostasis model assessment of β-cell function (HOMA-β), TNF-α and high sensitivity C-reactive protein (hsCRP). However, the propolis group had a considerable enhancement in the serum level of HDL-C in comparison with the placebo group. The therapeutic properties of Iranian propolis on decreasing serum insulin, postprandial blood glucose, inflammatory cytokines, and insulin resistance were seen. It also increases HDL-C concentrations in patients with T2D. Propolis treatment resulted in glycemic control. This might be due to decreasing intestinal absorption of carbohydrates, raising the level of glycolysis, suppression of glucose release in circulation from the liver, and inducing uptake of glucose by skeletal muscle cells via inducing the activation of insulin-sensitive glucose transporter (90).

Thus, the studies have demonstrated that propolis and its constituents have anti-diabetic effects by mechanisms such as reducing blood glucose, protective effect on beta cells, anti-oxidant, anti-inflammatory, inhibiting TNF-α expression, etc. (Table 5). 

**Table 1:** Summary of the effects of propolis and its constituents on obesity

Ref.	Study design	Results/Mechanisms	Dosage/Duration		Constituent
(50)	Humans/37 healthy volunteers	↑BWG/ ↑appetite of the volunteers, hepatic glycolysis, uptake of glucose by tissues through insulin-sensitive glucose transporter	1000 mg/60 days		Bee propolis
(34)	*In vivo*/Mice	↓BWG/ ↓adipogenesis ↑Energy expenditure	50 mg/kg/30 days		EEP
(39)	*In vivo*/Mice	↓BWG/↓absolute and relative fat mass in epididymal, retroperitoneal, or inguinal fat pads	20 mg per mouse per day/12 weeks		PPEE
(40)	*In vivo*/ Wistar rats	↓Weight of white fat tissue of the rats/↓PPARγ protein		0.5% (w/w) and 0.05% (w/w)/8weeks	Phenolic composition in propolis
(46)	*In vivo*/Mice	↓BWG/suppressing the ERK activity inhibiting the formation of adipose tissues		8% propolis/8 weeks	Propolis from New Zealand
(41)	*In vivo*/ C57BL/6N mice	↓Weight of visceral adipose and body weight gain/ ↓SREBP-1, SREBP-2 mRNA levels, and gene expression of FAS and ACAC activated by SREBP-1. →Down-regulation of FAS		0 mg/kg, 5 mg/kg, or 50 mg/kg twice daily/10 days	Brazilian propolis
(43)	*In vivo*/Mice	↓Level of adiposity, body weight gain, and adipogenesis ↑Energy expenditure /↓mRNA expression of lipogenic and adipogenic genes like C/EBPα, PPARγ, and FAS ↑mRNA expression of thermogenic and lipolytic genes such as PGC-1α, ATGL, PGC-1α, and ACSL		0, 0.02, 0.1, or 0.5% / 5 weeks	CAPE
(44)	*In vivo*/ C57BL/6J mice	↓Body fat accumulation ↓ Obesity ↓Risk of metabolic syndrome		5 mg/kg and 10 mg/kg/4 weeks	ArtC
(45)	*In vivo* /Rats	↓BW, BMI, AC/TC ratio, and adiposity index /↓Acute feed intake		25, 50, and 100 mg/kg/ 16 weeks	Chrysin
(42)	*In vivo*/ Female C57BL/6NRj wild-type mice	No effect on the BW and food intake		2.3 g/kg/10 weeks	GPSE
(91)	*In vivo*/ Sprague-Dawley rats	↓BW		100 mg/kg/21 days	Phenolic content of propolis
(92)	*In vivo* / C57BL/6 mice	↓BW/ ↓Food intake		100 mg/kg twice a week/five weeks	Brazilian green propolis ethanol extract
(79)	*In vivo*/ Male C57BL/6J mice	↓Obesity/changing the function and composition of gut microbiota in mice		1% and 2%/12weeks	EEP
(93)	*In vivo*/Female and male C57BL/6 6–mice	↓BW (male gender was more sensitive than female)/ reverses dysbiosis of gut microbiota in the HFD group, ↑Thermogenesis, ↑ BAT, and ↑Lipid metabolism in HFD mice.		150 and 300 mg/kg/9 weeks	CP
(94)	*In vivo*/ C57BL/6 Mice	↓Weight gain/↓feces weight ↓Fat absorption		2.0% (w/w)/ 12 weeks	Brazilian green propolis
(59)	*In vivo*/Rats	↓BW/↓adipocyte size ↓Leptin		200 mg/kg/4 weeks	Propolis
(95)	*In vivo*/Male C57Bl/6J mice	↓BWG/↓adiposity index in HFD mice, plasma levels of leptin, and the mRNA levels of TNF-α, CCL2, CCL5 ↑PPAR-α, MCAD, and LCAD, energy expenditure, and the oxidative program of fatty acid		4.5 mg of total polyphenols/mouse/day/12 weeks	poplar propolis extract powder
(96)	*In vivo*/ Streptozotocin-Treated Rats	↓Body weight loss		honey (1 g/kg/ day) and propolis (100 mg/day)/15 days	*Arbutus Unedo* Honey and Propolis
(97)	*In vivo*/Adult male Wistar rats	↓BW		150 mg/kg/day/30 days of 90 days	Red propolis
(98)	*In vivo*/Mice	↓obesity/↓CREB/CRTC2 transcriptional complex formation ↑Insulin sensitivity		Up to 100 μM/3 weeks	Artepillin C
(49)	*In vitro* in the stem cells-derived adipocytes	↓Lipid droplets/↓ROS ↑Expression of the HO-1 ↓Lipogenesis		10 μM/14 days	CAPE
(48)	*In vitro* test on 3T3-L1 cells	Induction of genes critical for differentiating of adipocyte including adiponectin Reversed the inhibitory effects of TNF alpha on adiponectin expression and adipocyte differentiation		(0–30 μg/ml) for 10 days	EERP
(43)	*In vitro*/3T3-L1 mouse preadipocyte cell line	↓Level of adiposity ↓Adipogenesis ↑Energy expenditure ↓mRNA Expression of lipogenic and dipogenic genes like C/EBPα, PPARγ, and FAS ↑mRNA expression of thermogenic and lipolytic genes such as PGC-1α, ATGL, PGC-1α, and ACSL		0, 0.02, 0.1, or 0.5% / 5 weeks	CAPE
(99)	*In vitro*/ Inguinal white adipose tissue (iWAT) of mice	Induction of brown-like adipocytes formation in iWAT → Release of energy via thermogenic UCP1 protein		10 mg/kg body weight (BW)/4 weeks(wk)	ArtC
(92)	*In vitro*/3T3-L1 adipocytes	↓BW/↑leptin expression in differentiated 3T3-L1 adipocytes ↓Food intake		100 mg/kg twice a week/five weeks	Brazilian green propolis ethanol extract
(45)	*In vitro*/ Adipocytes	↓AC/TC ratio, and adiposity index ,inhibitory effect on pancreatic LP, ↓acute feed intake		25, 50 and 100 mg/kg/ 16 weeks	Chrysin
(47)	*In vitro* *and In silico*	Anti-obesity/Lipase inhibitory effects		83 μM	Australian propolis compounds

↑: increase; ↓:decrease; BWG: body weight gain, ROS: reactive oxygen species, PPARγ: peroxisome proliferator-activated receptor gamma, ERK: extracellular-signal-regulated kinase, SREBP-1: Sterol regulatory element-binding protein 1, SREBP-2: Sterol regulatory element-binding protein 2, ACAC: acetylacetonate anion, TNF: tumor necrosis factor, FAS: fatty acid synthase, C/EBPα: CCAAT/Enhancer Binding Protein, PGC-1α: peroxisome proliferator-activated receptor-gamma coactivator 1α, ATGL: adipose triacylglycerol lipase, ACSL: long-chain acyl CoA synthetase, BMI: body mass index, AC/TC: abdominal circumference/thoracic circumference, LP: lipase, BW: body weight, HFD: high-fat diet, BAT: brown adipose tissue, iWAT: inguinal white adipose tissue, UCP1: uncoupling protein, EEP: ethanol extract of propolis, CAPE: caffeic acid phenethyl ester, PPEE: poplar propolis ethanolic extract, CRC: colorectal cancer, EERP: ethanolic extracts of red propolis, ArtC: artepillin c, GPSE: Brazilian green propolis supercritical extract, EEP: ethanol extract of propolis, CP: Chinese propolis, CREB: cAMP response element-binding protein, CRTC2: CREB regulated transcription coactivator 2, CCL2: chemokine (C-C motif) ligand 2, MCAD: medium chain acyl-CoA dehydrogenase, LCAD: long chain acyl-CoA dehydrogenase

**Table 2:** Summary of the effects of propolis and its constituents on hypertension

Constituents Dosage/Duration Results/Mechanisms Study design Ref.				
(52)	*In vivo*/ L-NAME induced hypertensive Spraque-Dawley rats	↓BP/↓TH ↓Biosynthesis of catecholamine	200 mg/kg/day/ 28 days	Propolis and CAPE
(53)	*In vivo*/ SHR	↓SBP/ vasodilation	10 mg/kg/28 days	Flavonoids
(54)	*In vivo*/ L-NAME induced hypertensive Wistar rats	↓BP and oxidative stress/ ↑CAT ↑NO ↓MDA Vasodilation	200 mg/kg/ the last 5 days from 15 days	Propolis
(55)	*In vivo*/ L-NAME induced hypertensive Sprague Dawley rats	Anti-hypertensive effect/↓ ADMA and NF‐κB ↑NO	Cape:50 μM/kg/day propolis :200 mg/kg/day / the last 14 of 28 days	Cape and propolis
(56)	*In vivo*/ L‑NAME induced hypertensive male albino rats	Hypotensive, and anti-oxidant effect / ↓TBARS, ↓conjugated dienes ↓Lipid hydroperoxides ↓Vitamin E ↓Vitamin C ↑ CAT ↑Superoxide dismutase ↑Glutathione peroxidase Vasodilation	25 mg/kg/4 weeks	Chrysin
(57)	*In* *vivo* test/ L‑NAME induced hypertensive male Wistar rats	↓Hypotensive and antioxidant effect/ ↓ MDA ↓CAT activity ↓ROS ↑NO Vasodilation	200 mg/kg/ the last 5 of 15 days	Propolis
(35)	*In vivo*/ High salt-induced hypertensive rats	Anti-oxidant, anti-inflammatory, and anti-hypertensive effect/ ↑eNOS and NO ↓IL-6 ↓TNF-α ↓Nox2 ↓Nox4 ↓ROS	100 mg/ kg/ the last 4 of 14 weeks	Chinese WSP
(58)	*In vivo*/ L‑NAME induced hypertensive rats	Anti-oxidant, anti-inflammatory, and anti-hypertensive effect/ ↓MDA ↓NF-κB ↓ TAS ↑CAT ↑ PON1 ↑total antioxidant status ↑NO Vasodilation	200 mg/ kg/28 days	Propolis
(59)	*In vivo*/High salt-induced hypertensive rats	↓SBP and DBP /↓LDL ↑HDL Diuretic effect Widening of the blood vessel	200 mg/kg/4 weeks	Propolis from Riau Archipelago, Lampung, and South Sulawesi
(51)	*In vivo*/ L‑NAME induced hypertensive Sprague-Dawley rats	↓BP, Anti-oxidant, and anti-inflammatory/ ↑PON1 (U/L) ↑TAS (mmol/L) ↓TOS (μmol/L) ↓OSI (ratio) ↓ADMA (ng/ml) ↓NF-κB(mmol/ml) ↑NO Vasodilation	Ethanolic extract of propolis: 200 mg/kg CAPE: 50 μM/kg/days /the last 14 of 28 days	EEP and CAPE
(97)	*In vivo*/ 5/6 renal ablation model hypertensive adult male Wistar rats	↓BP/ ↓Oxidative stress ↓inflammation renoprotective vasodilation	150 mg/kg/day/30 days of 90 days	Red Propolis
(100)	*In vivo*/ L‑NAME induced hypertensive rats	BP/↓TH ↓Biosynthesis of catecholamine ↑NO Vasodilation	200 mg/kg /the last 5 of 15 days	Propolis from Turkey
(101)	*In vivo*/SHR	↓SBP/-	5 mg/kg twice a day for 28 days	Brazilian propolis
(102)	*In vivo*/SHR	↓SBP and DBP/ ↑acetylcholine ↑Urine volume Vasodilation Anti-oxidant effect	0.5% propolis/7 days	Brazilian propolis
(103)	*In vivo*/Cadmium-induced hypertensive rats	↓ MBP/ ↓MDA ↑NO ↓Oxidative stress Vasodilation	10 μM/kg /15 days	CAPE
(104)	*In vivo*/L-NAME induced hypertensive rats	Anti-hypertensive effect/ ↓CAT ↓MDA ↓Oxidative stress ↑NO Vasodilation	200 mg/kg / the last 5 of the 15 days	Ethanolic extract of propolis
(105)	*In vivo*/ L-NAME induced hypertensive rats	Remarkable blood pressure reduction/↓Sympathoexcitation ↓oxidative damage (vascular and systemic) ↑Levels of NO in plasma anti-inflammatory and antioxidant properties through inhibiting the tumor necrosis factor receptor 1 up-regulation, phospho-nuclear factor kappa B, and vascular cell adhesion protein 1 and decreasing TNF-α	30 or 60 mg/kg/2 weeks	Galangin
(106)	*In vitro*	Anti-hypertensive effect/ ACE inhibitory effect	-	Propolis from Tunisia
(107)	*In vitro*	Anti-hypertensive effect/ ACE inhibitory effects	Between 65.49 mg GA/g and 228.40 mg GA/g	Propolis
(60)	Clinical/ a randomized controlled trial	Anti-oxidant and hypotensive effect/ ↓TBARS ↓ROS ↑ GSH ↑NrF2	3%/90 days	Total phenolic content of propolis

↑: increase; ↓: decrease; BP: blood pressure, TH: tyrosine hydroxylase, SBP: systolic blood pressure, CAT: catalase, NO: nitric oxide, MDA: malondialdehyde, ADMA: asymmetric dimethylarginine, NF‐κB: nuclear factor kappa b, TBARS: thiobarbituric acid reactive substances, ROS: reactive oxygen species, eNOS: endothelial nitric oxide synthase, IL-6: interleukin 6, TNF-α: tumor necrosis factor, Nox NADPH: oxidase, TAS: total oxidant status, PON1: paraoxonases, DBP: diastolic blood pressure, LDL: low-density lipoprotein, HDL: high-density lipoprotein, ACE: angiotensin-converting enzyme, GSH: glutathione, NrF2: Nuclear factor erythroid 2-related factor 2, OSI: oxidative stress index, TOS: total oxidative stress, MBP: mean blood pressure, CAPE: caffeic acid phenethyl ester, WSP: water-soluble propolis, EEP: ethanolic extract of propolis

**Table 3:** Summary of the effects of propolis and its constituents on atherosclerosis

Constituents	Dosage/Duration	Results/Mechanisms	Study design	Ref.
EECRP	/30 day 50 mg/kg	↑HDL-C,↓LDL, ↓Weight gain, ↓TG/ Antioxidant, Lipid profile regulation	*In vivo*/Mice feed with edible sunflower seed oil as HFD	(34)
EECP	160 mg/kg/d / 14 weeks	↓ Atherosclerotic lesions, ↓TG, ↓TC ,↓Non-HDL-C, ↓VEGF, ↓IL6,↑IL17/Anti-inflammatory, Modulation of lipid metabolism.	*In vivo*/ 16Male ApoE−/− mice were randomly divided into 2 groups fed a HFD	(65)
EECP	150 mg/kg/4 weeks-(0- 5.0) μg/ml/ 48 hour	↓RCT/↑ABCG1,↑ABCA1,↑HDL-C	*In vivo*-*in vitro*/12 mice were divided into two groups injected with 3H-Cholesterol-labeled and oxLDL-loaded Raw264.7 cells	(62)
PEP	250 mg/kg per day/4 weeks	↓TAG, ↓TC, ↓non-HDL-C, ↓Atherosclerotic lesions, ↑HO-1, ↓VCAM-1, ↓TGF-β /↑ HDL-C,↓CD36, ↑ABCA1	*In vivo*/ Mice (LDLr−/−, C57BL/6J) divided into two protocols, with the same five groups in each protocol , n=15 per group	(63)
PIN	10 mg/kg/d, 20 mg/kg/d / 14 w	↑EPCs/MNCs differentiation	*In vivo* and *in vitro*/ ApoE-/- mice fed with HFD, EPCs divided into three groups, n=12 per group	(108)
PCP	250 mg/kg	↓VEGF-A,↓Hif1a expression/ ↑miR-20b or ↑miR-106a	*In vivo*/50 Male mice (Ldlr-/-) fed an HCD were divided into 2 groups (n = 25)	(66)
EECP/TQ	75 mg/kg /5w	↓TBARS,↓TC,↓TG,↓LDL-C/Antioxidant	*In vivo*/16 Male New-Zealand white rabbits	(64)
EEP	125 and 250 mg/kg per day/6 weeks	protective effect on carotid restenosis in hypercholesterolemia rabbits,↓inflammation ,↓ oxidative stress/ ↓TLR4,↓NF-κB,↓blood lipid levels,↓ox-LDL,↓MDA,↑SOD,↑No	*In vivo*/24male New Zealand white rabbits were randomized into four groups (n = 6)	(109)
EEBP	0.5% (w/w)/ 8 weeks	↓Thrombotic tendencies/↑PAI-1	*In vivo*/Mice	(110)
AEBP	1–4 μg/ml	↓LDL oxidation,↓PCD/antiapoptotic , ↓Copper-mmLDL	*In vitro*/ Human peripheral blood mononuclear cells exposed to mmLDL	(111)
EECP/EEBP	12.5 𝜇g/ml/12 hr and 24 hr	↓ROS,↓NF-𝜅B/↓ox-LDL	*In vitro*/ HUVECs treated with ox-LDL	(67)
EEMSP	20 μg/ml/ 48 hr	↓IL-1β,↓TNF-α, ↓CE, ↓Lipid droplet/ ↓MAPK	*In vitro*/ THP-1 derived macrophages treated with ox-LDL	(61)
EEBP	Prpolis(0.001 - 0.005%) or its constituents (20 μM) / 12h	↓PAI-1/↓Inflammatory reactions ↓NF-κB	*In vivo* and *in vitro*/Mice-HUVECs	(112)
CAPE-NO2	1.5–12μM	↓Platelet aggregation/↓5-HT,↓COX-1enzyme activity,↑NO,↑cGMP	*In vitro*/ Abdominal aorta platelet of rat	(113)
Isoflavone (3S)-vestitol,	0.37-0.55 µM /48h	↓Inflammatory reactions/↓NF-κB , ↓IL-1β, ↓IL-1α, ↓G-CSF,↓ IL-10 ,↓GM-CSF, ↓Icam-1, ↓Wnt5a ,↓Mmp7,↑Socs3 , ↑Apoe,↑Igf1,↑Fgf10	*In vitro*/ Peritoneal macrophages of C57BL6 mice	(114)

↑: increase; ↓:decrease; TG: Three glisride, EECRP: ethanolic extract of Croatian propolis, PEP: polyphenol extract of propolis, TQ: thymoquinone, AEBP: aqueous extract of Brazilian propolis, EEMSP: Ethanolic extract of Malaysian stingless bee propolis, VEGF: vascular endothelial growth factor, HUVECs: human umbilical cord endothelial cells, EECP: ethanoic extracts of Chinese propolis, EEBP: ethanoic extracts of Brazilian propolis, RCT: reverse cholesterol transport, MAPK mitogen-activated protein kinase, PIN pinocembrin, EPCs endothelial progenitor cells, MNCs mononuclear cells, PCP polyphenolic-rich extract from Chilean, PCD: programmed cell death, mmLDL minimal modifiziertes LDL, EERP ethanolic extracts of Brazilian red propolis, PAI-1: plasma plasminogen activator inhibitor 1, CAPE-NO2: nitro derivative of caffeic acid phenethyl ester, cGMP: cyclic guanosine monophosphate, ROS: reactive oxygen species, NF-𝜅B: nuclear factor kappa-light-chain-enhancer of activated B cells, TC: total cholesterol, ox-LDL: oxidase low-density lipoprotein, LDL: low-density lipoprotein, IL: interleukin, HDL: high-density lipoprotein, TGF: transforming growth factor beta, TNF: tumor necrosis factor, PAI: plasminogen activator inhibitor, PPAR: peroxisome proliferator-activated receptor, LXR: liver X receptor, Cox: cyclooxygenase, VCAM: vascular cell adhesion molecule 1, NO: nitric oxide, 5-HT: 5-hydroxytryptamine, ABCTA1: ATP-binding cassette transporter A1, TBARS: thiobarbituric acid reactive substances, Hif-1a: hypoxia-inducible-factor 1a, miR microRNAs, CE: cholesterol ester, TAG: triacylglycerols, ABCG1: ATP-binding cassette transporter G1, CD36: cluster of differentiation 36, HO-1: heme oxygenase 1, HFD: high-fat diet, HCD: cholesterol-enriched hypercholesterolemic diet, TQ: thymoquinone, LPS: lipopolysaccharide, DMEM: dulbecco’s modified eagle medium, FBS: fetal bovine serum, G-CSF: Granulocyte colony-stimulating factor , GM-CSF: granulocyte-macrophage colony-stimulating factor, ICAM: intercellular adhesion molecule, wnt: Wingless/Integrated, MMP: matrix metalloproteinase, SOCS: Suppressor of cytokine signalling proteins, APOE: apolipoprotein E, IGF: insulin-like growth factor , Fgf: fibroblasts growth factor, EEP: ethanolic extract of propolis, MDA: malondialdehyde, SOD: superoxide dismutase , TLR: toll-like receptor

**Table 4:** Summary of the effects of propolis and its constituents on dyslipidemia

Ref.	Study design	Results/ Mechanisms	Dosage/Duration	Constituents
(74)	*In vivo*/rats	↓TC, TG,↑HDL	50 , 100, and 200 mg/kg/day	propolis
(115)	*In vivo*/12-month-old, male, White rabbits	↓TC, TG,↑HDL	150 mg/kg/10 weeks	Egyptian propolis and vitamin E
(36)	*In vivo*/Adult male Wistar Albino rats	↓ TC,TG, and VLDL-C/ ↑HDL-C/ up-regulation of ABCA1 gene expression	100 mg/kg/day/30 days	Malaysian Propolis
(75)	*In vivo*/Diabetic mice	↓TG, TC, and LDL-C ↑ HDL-C/↑PPARα	15 and 30 mg/kg BW	CAPE
(93)	*In vivo*/Female and male C57BL/6 mice	In male mice: ↓ TC, HDL, and TG but little effect on LDL In female mice: no alteration	150 and 300 mg/kg/9 weeks	Chinese Propolis
(116)	*In vivo*/Normal and diabetic rats	No effects in non-diabetic rats/↓TG, TC, VLDL and LDL-C, ↑HDL-C in diabetic rats	100 and 200 mg/kg BW/16weeks	Moroccan propolis and bee pollen
(117)	*In vivo*/Male Sprague–Dawley rats	↓TC	10 mg per 100 g BW	Chinese Propolis and Brazilian Propolis
(65)	*In vivo*/ male ApoE-knockout mice	↓TC, TG, and non-HDL-C,↓ no dramatic effect on HDL-C	160 mg/kg/day/14 weeks	EEP
(41)	*In vivo*/ C57BL/6N mice	↓Liver and serum TG, TC, non-esterified fatty acids/↓SREBP-1, SREBP-2 mRNA levels	0, 5, or 50 mg/kg twice daily for 10 days	Brazilian propolis
(37)	*In vivo*/Adult male Wistar rats	↓TC, TG, LDL, and VLDL/↑HDL	50 mg/kg BW/15 days	Moroccan propolis
(40)	*In vivo*/ Wistar rats	↓TC, TG in plasma and liver in the high group/↓SREBP-1,HMGR/↑PPARα/	0.5% (w/w) and 0.05% (w/w)/8 weeks	Propolis
(118)	*In vivo*/Adult Albino mice	Non-dramatic change in lipid serum levels compared with control group. Serum HDL values reduced non-dramatically as compared with control group	50 mg/kg BW	American propolis
(73)	*In vivo*/Male Wistar rats	↓TC,TG and LDL-c/↑HDL-C/ anti-inflammatory effects	20 mg/kg/30 days	Chrysin
(119)	*In vivo*/ Male Wistar rats	↓TG and TC/ Inhibitory activity on the HMGR	20% concentration of the ointment	Green and Red propolis
(72)	*In vivo*/Wistar Swiss albino male rats (Rattus rattus)	↑HDL, ↓LDL, TC, and TG/via inhibition of free radicals	200 mg/kg/15days	Turkish propolis
(69)	*In vivo*/Sprague Dawley rats	↓TC, LDL/ no significant differences in TG and HDL levels between MSFF and HCD groups/inhibition of lipid peroxidation, HMGR and ACAT2 activities	50,100, and 200 mg/kg/one dose	MSFF
(120)	*In vivo*/ Male Wistar rats	No alterations in HDL level in different treatments	1, 3, and 6 mg/kg/day/30,90,150 days	Propolis, EEP and WEP
(34)	*In vivo*/Male C57BL/6N mice	↑HDL-C, ↓LDL-C, TC and TG	50 mg/kg/30 days	EEP
(121)	*In vivo*/Adult female C57BL/6 mice	↓TC/Preventing the augmentation of TC	10 mg/kg/one dose	Chrysin
(71)	*In vivo*/Rats	Low changes in the concentration of LDL, HDL, and VLDL/↓TC, TG/ Lipase stimulation/Inhibition of HMGR	1 and 2% propolis in cholesterol diet/2 weeks	Saudi propolis
(76)	Human study/Patients with T2D	↓TG, LDL	300 mg three times a day/12 weeks	Propolis

↑: increase; ↓: decrease; T2D: type-2 diabetes, LDL-c: low-density lipoprotein cholesterol, HDL-c: high-density lipoprotein cholesterol, VLDL-C: very-low-density lipoprotein cholesterol, TC: total cholesterol, TG: triglyceride, EEP: ethanolic extract of propolis, WEP: water extract of propolis, CAPE: caffeic acid phenethyl ester, MSFF: mixtures of selected functional foods, BW: body weight, HCD: high cholesterol diet, ACAT2: acetyl-CoA acetyltransferase 2, HMGR: 3-hydroxy-3-methyl-glutaryl-CoA reductase, SREBP: sterol regulatory element-binding protein, PPARα: peroxisome proliferator-activated receptor

**Table 5:** Summary of the effects of propolis and its constituents on diabetes

Constituents Dosage/Duration		Results/ Mechanisms	Study design	Ref.
Water-soluble derivatives and ethanoic extracts of chinese propolis	-/ 8 weeks	↓FBG, ↓FRU,↓MDA, ↓NO, ↓NOS,↓TC,↓TG, ↓LDL-C, ↓VLDL-C,↑HDL-C, ↑SOD	,72 rats divided into six groups	(122)
Freeze-Dried Extract of Malaysian Propolis	300 mg/kg b.w/4 weeks	↓FBG,↓Final body weight,↑Absolute liver weight,↓Serum markers of Hepatic lesion,↓LDH activity,↑SOD, ↑CAT,↑GPx,↑GST,↑GR,↑GSH,↓MDA, ↑TAC/↓ 1-scavenging ROS or/and FBG	*In vivo*/ STZ-induced diabetic rats were randomly divided into 5 groups (n = 6 pre group )	(83)
Chrysin	40 mg/kgbw/16 weeks	↑GSH, ↑GPx, ↑GR, ↑SOD,↓MDA,↓Serum creatinine, ↓BUN, ↓Proteinuria,↑The creatinine clearance,↓TNF-α, ↓NF-κb(p65), ↓TGF-β, ↓Fibronectin , ↓Collagen-IV/ ↓TNF-α, ↓NF-κB activation,	*In vivo*/ HFD/STZ-induced diabetic rats ,24 rats were randomly divided into four groups (n=6 pre group)	(84)
CAPE	30 mg/Kg/ 21 days (excluding debits induction)	↓Bodyweight, ↓Blood glucose, ↓Water intake, and urine excreted, ↑Plasmatic insulin, ↑RSH,$↓{\mathrm{NO3}}^{-},↓{\mathrm{NO2}}^{-}$,↓ADMA, ↓LOOH,↑GGCL, ↓iNOS,↑DDAH-1/ Inhibit lipid peroxidation, Regulating antioxidant enzyme-related proteins	*In vivo*/STZ induced diabetic rats were divided into 4 groups (n=6 pre group)	(123)
BP and CP	100 mg/kg/8 weeks	↓MDA, ↑GSH-px / Inhibiting lipid peroxidation, enhancing antioxidant enzymes activities	*In vivo*/Diabetic rats	(124)
CAPE	10 µmol/kg/8 weeks	↓MDA,SOD,CAT, ↑GSH-px/Antioxidant effect	*In vivo*/Male Sprague–Dawley rats	(125)
Ethanol-soluble derivative of propolis	100 mg/kg BW/1 month	Enhancing lymphocyte proliferation and chemotaxis towards CCL21 and CXCL12/antioxidant effect	*In vivo*/12-week-old male BALB/c Mice	(126)
Malaysian Propolis and Metformin	300 mg/kg BW/4weeks	↓FBG level /Inhibiting α-glucosidase activity, Pancreatic β-cell regeneration, ↑Insulin secretion, ↓Hepatic gluconeogenesis	*In vivo*/Male Sprague-Dawley rats	(127)
EEMP	300,600 mg/kg/day/4 weeks	↓Total food intake, FBG, ↑BWG /Protective effect on β cells, Stimulating pancreas to secrete more insulin, inhibiting glucose release from the liver, antioxidant property	*In vivo*/Female Sprague Dawley rats	(87)
Moroccan propolis	50,100 mg/kg/BE/daily for 15 days	↓Abnormal liver and kidney function, ↓Lactic acid dehydrogenase, ↑BW/Phenols, and flavonoids content, Inhibiting α-glucosidase and α-amylase activities	*In vivo*/Adult male Wistar rats	(37)
Propolis	10, 90 mg/kg/7days	No effects after diabetes establishment	*In vivo*/Male Wistar rats	(128)
Malaysian propolis	300 mg/kg/day/4 weeks	↓Fetal blood glucose, /Reducing oxidative stress (Antioxidant effect)	*In vivo*/ Streptozotocin-induced diabetic female Sprague Dawley rats	(85)
EEP	100, 200 mg/kg/day/6 weeks	↓Serum glucose levels, kidney weight, and MDA/Antioxidant effect	*In vivo*/male adult Wistar rats	(86)
EECP	100 mg/kg/day/ twice daily in the morning and the afternoon for 12 weeks	Reducing oxidative stress, Inhibiting photoreceptor cell degeneration, Regulating BRB permeability, Preventing retinal thickening and the loss of tight junction proteins, ↓FBG, HbA1c/-	*In vivo*/ Male brown Norway rats	(129)
TGPE	183.9 and 919.5 mg/kg/day/8 weeks	↓Development T2D, Severity of β-cell failure, ROS/Free radical scavenging activity, anti-inflammation effects, anti-oxidant effects, and balancing lipid metabolism	*In vivo*/Male Sprague Dawley rats	(130)
Baccharin Isolated from Brazilian Green Propolis	10 or 50 mg/kg/5 weeks	↓blood glucose, ↓HOMA-IR values, ↑adipocyte differentiation/↑PPARγ	*In vivo*/ ob/ob mice were divided into 3 groups (n = 9–10) based on body weight and blood glucose levels.	(109)
CP	Effect 900 mg/day/18 weeks	No significant difference between the groups in serum glucose, glycosylated hemoglobin, insulin, aldose reductase, or adiponectin, ↑ GSH, flavonoids, and polyphenols, ↓ Serum lactate dehydrogenase activity/Antioxidant	Human study/ Patients with T2D	(88)
Iranian Propolis	↓Glycosylated hemoglobin (HbA1c), insulin, HOMA-IR, HOMA-β, ALT, AST, and BUN/ Inhibition of glucose release in circulation from the liver, ↓Intestinal absorption of carbohydrate, ↑Glycolysis, and utilization of glucose in the liver, Sensitive glucose transporter 500mg twice daily/90days	↓Glycosylated hemoglobin (HbA1c), insulin, HOMA-IR, HOMA-β, ALT, AST, and BUN/ Inhibition of glucose release in circulation from the liver, ↓Intestinal absorption of carbohydrate, ↑Glycolysis, and utilization of glucose in the liver, Sensitive glucose transporter	Human study /Patients with T2D	(90)
Iranian Propolis	900 mg/d/ one year	↓FBG, ↓HbA1c,↓TC, ↓LDL/ ↓Expression and activity of the glucose 6 phosphatase enzyme	Human study/Double-Blind, placebo-controlled clinical trial, 66 patients were randomly divided into two groups intervention and placebo	(76)
EEBGP	900 mg daily/ 18 weeks	↑Serum GSH, ↑Total polyphenols, ↓Serum carbonyls, ↓LDH, ↓TNF-α/-	Human study/Type 2 diabetes patients	(131)
Iranian propolis	1500 mg/d / 8 weeks	↓Insulin reasistance, ↓HbA1c, ↓FBS /Antioxidant, ↑Total antioxidant capacity, ↑SOD, ↑GPx	Human study/A randomized, double-blind, placebo-controlled Study,62 patients were randomly assigned to one of the intervention or placebo group (n=31)	(132)
Iranian propolis	1500 mg/ 8 weeks	↓Ox-LDL, ↓CAT Activity, ↓FRU	Human study/A randomized, double-blind, placebo-controlled Study,62 patients were randomly assigned to one of the intervention or placebo group (n=31)	(89)

↑: increase; ↓: decrease; FBS: fast blood sugar, VLDL: very low-density lipoprotein, FRU: fructosamine, BW: body weight, MPOs: myeloperoxidase, GSH: glutathione, TAC: total antioxidant capacity, GR: glutathione reductase, GST: glutathione-S-transferase, LDH: lactate dehydrogenase, WSDP: water soluble derivative of propolis, Cox: cyclooxygenase, PMN: polymorphonuclear leu-kocyte, MMP-9: matrix metalloproteinase-9, LOOH: lipid hydroperoxide, RSH: non-proteic thiol groups, DDAH-1: dimethylarginine dimethylaminohydrolase-1, HbA1C: hemoglobin A1c, BP: Brazilian propolis, CP: Chinese propolis, EECP: ethanol extract of chinese propolis, GBM: glomerular basement membrane, TGPE: Taiwanese green propolis ethanol extract, EEP: ethanolic extracts of propolis, superoxide, EEBGP: Ethanolic extract of brazilian green propolis, GPx: glutathione peroxidase, FBG: fasting blood glucose, malonaldehyde, NO: nitrogen oxide, NOS: nitric oxide synthase, TC: total cholesterol, TG: triglyceride, LDL: low-density lipoprotein, HDL: high-density lipoprotein, CAT: catalase, ROS: reactive oxygen species, BUN: blood urea nitrogen, TNF: tumor necrosis factor, NF-𝜅B: nuclear factor kappa-light-chain-enhancer of activated B cells, TGF-β: transforming growth factor beta, ox-LDL: oxidase low-density lipoprotein, CAPE: caffeic acid phenethyl ester, BWG: body weight gain, IL: interleukin, BRB: blood-retinal barrier, ALT: alanine aminotransferase, AST: aspartate aminotransferase, T2D: type 2 diabetes, ROS: reactive oxygen species, HFD: high-fat diet, STZ: streptozotocin, HOMA-IR: homeostasis model assessment-insulin resistance, HOMA-β: homeostasis model assessment of β-cell function, EEMP: ethanol extract of Malaysian propolis, PPARγ: Peroxisome proliferator-activated receptor gamma

## Conclusion

CVDs are some of the major causes of death around the world. Hypertension, obesity, atherosclerosis, diabetes, and dyslipidemia are considered risk factors for CVDs. 

Studies have demonstrated that different kinds of propolis and its constituents have anti-obesity effects through mechanisms such as reducing adipogenesis, lipid droplets, absolute and relative fat mass in epididymal, retroperitoneal, or inguinal fat pads, PPARγ, feces weight, fat absorption, adipocyte size, leptin, SREBP-1, SREBP-2 mRNA levels, gene expression of FAS, acute feed intake and elevating expenditure of energy, thermogenesis, brown-like adipose tissue (BAT), lipid metabolism, inhibitory effect on pancreatic LP and ERK activity, anti-oxidant, anti-inflammatory, and induction of brown-like adipocytes. Moreover, propolis and its constituents have anti-hypertensive effects by mechanisms such as increasing the NO level, diuretic, anti-oxidant, ACE inhibitory, reducing TH activity, biosynthesis of catecholamine, lipid hydroperoxides, and inflammation. 

Propolis also has anti-atherosclerosis effects by the mechanisms such as anti-oxidant, reversing cholesterol transfer, change in lipid profile, anti-inflammatory, reversing the imbalance between NO and ET, reducing neovascularization, and preventing endothelial dysfunction.

Propolis and its constituents also have antidyslipidemic effects by mechanisms such as HMG- CoA reductase inhibitory, decreasing SREBP-1 and SREBP-2 mRNA levels, chrysin’s anti-inflammatory effects, lipase stimulation, up-regulation of ABCA1 gene expression, and reducing lipid peroxidation. Besides, propolis and its constituents have antidiabetic effects through mechanisms such as reducing the FBS level, inhibition of lipid peroxidation, lipoxygenase and the COX pathway, glucose release from the liver, α-glucosidase and α-amylase activities, sensitive glucose transporter, enhancing anti-oxidant enzymes activities, decreasing intestinal absorption of carbohydrate, MPOs activity, expression and activity of the glucose 6 phosphatase enzyme, stimulating macrophages and PMN, anti-inflammatory activity, and increasing insulin secretion. In summary, based on this review, propolis and its constituents can be effective in the treatment and prevention of cardiovascular risk factors including obesity, hypertension, atherosclerosis, diabetes, and dyslipidemia.

Due to limited clinical trials, more human studies are needed to confirm the results and mechanisms.

## Authors’ contributions

A K, AH H, and AR M provided data curation, investigation, writing, and the original draft; BM R critically revised the paper and supervised the research; H H conceived the study, designed and supervised the research, and edited the manuscript. 

## Conflicts of Interest

None.
